# Interventions Through Music and Interpersonal Synchrony That Enhance Prosocial Behavior: A Systematic Review

**DOI:** 10.3390/ejihpe15030029

**Published:** 2025-02-28

**Authors:** Mireia Pardo-Olmos, Manuel Martí-Vilar, Sergio Hidalgo-Fuentes, Javier Cabedo-Peris

**Affiliations:** 1 Basic Psychology Department, Faculty of Psychology and Speech Therapy, Universitat de València, 46010 Valencia, Spain; miparol@alumni.uv.es (M.P.-O.); sergio.hidalgo@uv.es (S.H.-F.); 2 Faculty of Health Sciences, Valencia International University VIU, 46002 Valencia, Spain

**Keywords:** prosociality, music, interpersonal synchrony, prosocial behavior, musical intervention

## Abstract

Prosociality is essential in human interactions, characterized by voluntary behaviors aimed at benefiting others. Promoting such behaviors is indispensable for human relationships. Studies have demonstrated positive effects of interpersonal synchrony interventions through musical sessions. Some authors suggest that the joint creation of music facilitates prosocial behaviors (PB). This review aims to determine whether evidence supports the idea that participation in a musical interpersonal synchrony experience improves PB. A systematic review of 12 selected articles was conducted. The search was carried out in databases such as WoS, Scopus, PubMed, Dialnet, Elsevier, and Eric. Results indicate that music, as a tool, creates an optimal environment for mimesis and group participation, which can enhance prosociality. Controlling variables such as sample size, population type, measurement instruments, techniques, and session design is necessary for future research. It is concluded that interpersonal synchrony through music appears to improve prosociality, although more robust studies are required.

## 1. Introduction

### 1.1. Prosociality

Prosociality is a complex term that has been extensively studied by researchers, generating debates regarding how this construct is measured and what its components are. They could not reach a definitive conclusion. The common feature of all definitions is that prosocial behavior reflects acts of “positive” social behavior towards the others (Pfattheicher et al., 2022). Prosocial behavior promotes personal well-being (Aknin et al., 2019) and the establishment of positive interpersonal relationships (Palomar & Victorio, 2018). Prosocial behavior can be defined as voluntary actions (Eisenberg & Fabes, 1998) that aim to meet another person’s physical and emotional support needs (Benson et al., 2006). It is adopted to care for, assist, confront, and help others (Caprara et al., 2005). As noted by Hart and Hart (2023), multiple concepts in the literature fall within the domain of prosociality, including helping behaviors, kindness, altruism, empathy, compassion, sympathy, and social support, among others.

Prosociality is so linked to altruism that the two concepts are difficult to distinguish. The motivations for prosocial behavior can be classified into two types: non-altruistic, associated with selfishness or seeking others’ approval, and altruistic, related to empathy, compassion, and caring for others (Botella & Montesinos, 2016). Consequently, there is a division in definitions that distinguishes between prosocial and altruistic behavior and those that do not (Martínez-Cano et al., 2019). It is considered that, for an act to be altruistic, it must meet the following conditions: the behavior must be voluntarily performed, the recipient must benefit in some way from the benefactor’s action, and the person performing the act must incur some cost to benefit the other (Moñivas, 1996).

The abundance of definitions indicates that prosociality is a multidimensional construct that can manifest in numerous forms (Hart & Hart, 2023). Auné et al. (2014) discuss strict and loose descriptions of prosocial behavior. Strict definitions require extensive criteria for a behavior to be considered prosocial, while loose definitions are simpler and have fewer requirements. On the one hand, a strict definition describes prosocial behavior as actions that benefit another person, groups, or social goals and increase the likelihood of generating positive, high-quality, and supportive reciprocity in subsequent interpersonal or social relationships while safeguarding the identity, creativity, and initiative of the people or groups involved (Roche-Olivar, 1998). On the other hand, a loose definition considers prosocial behavior as any social behavior that aims to benefit another, with or without altruistic motivation (González-Portal, 2000).

In summary, each prosocial behavior is unique, as it places specific demands on individuals. Despite its uniqueness, it is vital for the development and maintenance of harmonious relationships (Ilari et al., 2020). It addresses current social coexistence issues and improves quality of life, which is also linked to psychological adjustment (Cuadra-Martínez & Salgado-Roa, 2020). While its teaching is crucial, educational contexts often prioritize content learning (Arias-Gallegos, 2015). Given humanity’s inherent social nature, fostering tolerance, respect, healthy coexistence, and active citizenship is essential. By exploring effective and adaptable interventions and tools, prosociality can be promoted within the population.

### 1.2. Musical Interventions

Betés de Toro (2000) explains that music exerts effects at physiological, emotional, spiritual, cognitive, and social levels. Numerous benefits of employing music in educational and therapeutic contexts have been described (García-García & Rubio-Belmonte, 2020), such as its use with Alzheimer’s patients and pregnant women. Several studies have demonstrated that music can reduce anxiety and the sensation of pain. Music has been observed to reduce the need for medication, stimulate the brain, foster a positive mental attitude, and help with relaxation (Correa, 2010).

Music is present in all human cultures (Mehr et al., 2019). Its use can be found in traditional cultures for pragmatic reasons ((Kirschner & Tomasello, 2010); cited in Kirschner & Tomasello, 2010), integrated into ritual ceremonies such as worship, weddings, funerals, or preparations for hunting or combat (Clayton, 2009; Dissanayake, 2006; cited in Kirschner & Tomasello, 2010). Music has the ability to create emotional states, which are considered essential for maintaining group identity (Alaminos-Fernández, 2021). Betés de Toro (2000) asserts that music is accessible to all individuals and communities, is flexible, provides security, influences quality of life, and has a symbolic and non-verbal language that reaches everyone. Music is connected to societies, cooperation, and cohesion. It offers countless possibilities for expression, both individually and in groups (Blasco, 2016). The implementation of music-based therapies is widely known to yield numerous benefits (Lin et al., 2023; Thaut et al., 2021; Tramontano et al., 2021).

When discussing musical interventions, two commonly confused concepts must be differentiated: music education and music therapy. Music education is a structured process based on curriculum-described content, using music as an end in itself, with general and universal objectives designed to improve the quality of musical performances under a teacher’s guidance. In contrast, music therapy is a discipline where music is used as a means to produce changes among people; it has individualized objectives and is guided by a therapist. The literature includes studies employing these types of tools, highlighting the importance of distinguishing between them. In community settings, the term Social Music Therapy is used, which Schwabe and Haase (1998) define as a discipline that develops social competence, improves perceptual capacity and stimulates and promotes emotional processes through musical means that foster a sense of community, activating and motivating communication in children. Music, therefore, is an art form that represents a clear potential candidate to have an influence on social capacities due to its ability to evoke emotional states. Also, it can make listeners relate to the thoughts and emotional states of the performer (McDonald et al., 2022).

### 1.3. Prosociality and Music

Numerous studies report positive effects of engaging with music. Making music can enhance empathy (Rabinowitch et al., 2013) and promote helping behavior (Kirschner & Tomasello, 2010). Additionally, music is a culturally developed tool to foster group cohesion and commitment, which increases prosocial cooperation within the group (Hurón, 2001). A key feature of musical intervention is the synchronizing of interpersonal movements, including both vocal and body synchronization. Collective music creation has been observed to provide opportunities for individuals to engage in imitation and interpersonal coordination within a safe space, generating a sense of unity (Rabinowitch & Meltzoff, 2017). Furthermore, other studies suggest that music increases levels of cooperation, calmness, and positive affect such as smiling and laughing (Schellenberg et al., 2015).

Wan and Zhu (2021) demonstrated that music creation affects prosocial behavior in 5-year-old children. Similarly, Beck and Rieser (2022) found comparable results in preschool children (ages 3 to 5), who displayed more helping behavior towards the researcher after participating in a musical activity. However, other authors, such as Kirschner and Ilari (2014), did not find associations between brief percussion sessions and preschoolers’ prosocial skills towards the researcher after a musical intervention.

There are multiple inconsistent results in the literature, as has been seen. Studies examining the potential links between music education and prosocial behavior have mostly focused on children (Ilari et al., 2020). Studies on adults are scarcer. However, the association between shared musical activities and prosocial behavior has been found in studies involving both adults and children (Ilari et al., 2020). For example, Anshel and Kipper (1988) found that Israeli adult men cooperated better in the prisoner’s dilemma game and scored higher on a trust questionnaire after a group singing lesson. Nonetheless, Alemán et al. (2016) did not find the effects of the “El Sistema” program on prosociality in a large randomized controlled trial conducted with Venezuelan students. It highlights continued discrepancies in results.

The aforementioned studies are based on the idea that prolonged participation in forms of music-making can foster cooperation and strengthen the sense of belonging and group affiliation. These may lead to prosocial development (Graves, 2019). Through interpersonal synchrony, individuals can align their actions and transform them into a reward-like signal. It can affect prosocial tendencies, specifically towards those they have acted in synchrony with (Kokal et al., 2011). The experience of continuously coordinating to achieve shared goals likely strengthens the sense of shared intentionality and interdependence, thereby increasing prosociality. According to Buren et al. (2021), synchrony, along with having a shared goal, may be part of the explanation for improvements in prosocial behavior.

### 1.4. The Current Study

The main objective of this review is to determine whether evidence exists that participation in a musical interpersonal synchrony experience contributes to improving prosocial behavior among the general population. More specifically, it focuses on data suggesting that an experience of coordination and synchrony, both with others and individually, enhances prosocial behavior. Consequently, it is expected that the results will align with this premise, showing that participants who have experienced a situation under conditions of interpersonal synchrony through music display a higher level of prosocial behavior. Additionally, the second objective aims to examine and detect potential modulators in the selected articles that could be causing differences in the research findings. Using the PICO approach, the following research question (RQ) was proposed: Which effect will the musical interpersonal synchrony have on prosociality scores among individuals?

## 2. Materials and Methods

A systematic review was conducted following the criteria of Preferred Reported Items for Systematic Reviews and Meta-Analyses (PRISMA) in their 2020 Declaration (Page et al., 2021). The checklist provided by PRISMA was followed to ensure rigor in the proceeding (see Appendix A). Registration has been requested in the International Prospective Registers of Systematic Reviews (PROSPERO) and a response is pending.

### 2.1. Information Sources and Search Strategies

Information was collected from scientific literature related to the topic. A search was performed in 2023, starting in February and finishing in May. To this aim, the databases of Web of Science (WoS), Scopus, PubMed, Dialnet, Elsevier, and ERIC were used. Only the published reports written in English or Spanish until December 2022 were included. A search strategy combining specific keywords to address the topic was developed. It is versed as follows: (music) AND (prosocial*). This process was conducted with the help of Covidence© https://www.covidence.org/, a software designed to help with conducting systematic reviews. This tool also helped to ensure interrater reliability between two of the researchers who contributed to this study.

### 2.2. Eligibility Criteria and Selection Process

Inclusion and exclusion criteria were used to facilitate the eligibility of the reports included in this review. The inclusion criteria were (a) documents written in English or Spanish; (b) empirical research articles with a quantitative method; (c) double-blind reviewed studies; (d) research conducted on humans, regardless of their age; (e) documents analyzing the relationship between musical intervention and prosocial behavior or related attitudes, such as empathy, cooperation, helping, sympathy, or other related markers; (f) articles published from the earliest available database records on the subject until December 2022; (g) studies in which synchronous movements through music were reported.

The selected exclusion criteria were (a) books, doctoral theses, editorials, conference papers, clinical case studies, literature reviews, and other non-quantitative research; (b) articles using interpersonal synchrony without the presence of music; (c) studies involving participants with psychological disorders.

### 2.3. Data Collection Procedures and Critical Approaches

A protocol to systematically extract the characteristics of the studies was developed. This process was conducted on each of the studies included in the review. The characteristics were coded as follows: authorship and publication year; country where the study was developed; sample’s age, sample’s size, and a brief description of it; type of study design; assessed variables and their interactions’ effect size between them; measurement instruments used for evaluation; number of the intervention sessions; musical techniques and dynamics; and results regarding the music and prosocial behavior.

The Appraisal tool for Cross-Sectional Studies (AXIS) was used to perform bias assessment. This is a critical appraisal (CA) tool created by experts in 2016. It is the first CA tool for evaluating this type of evidence, which can be incorporated into systematic reviews, guidelines, and decision-making processes (Downes et al., 2016). This instrument is composed of 20 assessment items or questions related to sections of the introduction, method, results, conclusions, and other aspects (see Appendix B). Regarding the assessment content of the questions, seven are related to reporting quality (1, 4, 10, 11, 12, 16, and 18), seven are related to studying design quality (2, 3, 5, 8, 17, 19, and 20), and the remaining six address the potential introduction of bias in the study (6, 7, 9, 13, 14, and 15).

## 3. Results

### 3.1. Study Selection and Characteristics

**Figure 1:**
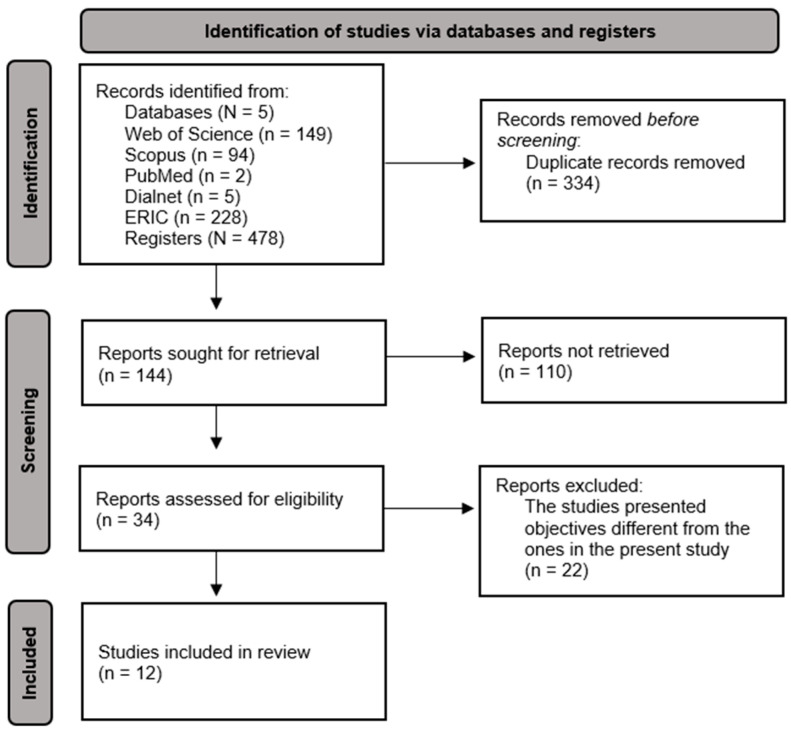
Flow diagram according to PRISMA guidelines.

After the initial screening, where duplicate documents were removed and abstracts were read, only 34 documents were deemed suitable. Subsequently, a deeper analysis was conducted over the full text. Finally, 22 of them were excluded because they addressed objectives that differed from the present study’s aim. This last screening led to 12 documents that were considered to meet the inclusion criteria and were selected for this review. This study selection process can be seen in Figure 1.

As detailed before (see Section 2.3), the most representative information of each study included in the review was extracted. The articles were published in various countries worldwide, including Australia, Canada, China, Colombia, Germany, the Netherlands, Spain, and the United States. Each country was represented in one of the articles, except for Germany and China, which accounted for 16.67% (n = 2), and the United States, which represented 25% (n = 3) of the selected articles. Table 1, detailing the characteristics of the articles, is presented below.

### 3.2. Synthesis of Results

This section includes both sociodemographic characteristics of the samples included in the study and methodological aspects of their analyses. Also, the results of the CA are presented.

#### 3.2.1. Sociodemographic Characteristics of the Samples

The 12 studies included a total of 3755 participants. The number of participants in the studies ranged from 18 to 3031, with an average sample size of 312.92. Of these, 91.67% (n = 11) of the articles had between 18 and 124 participants, with an average sample size of 65.82, whereas one article included a much larger sample of 3031 participants (Williams et al., 2015).

Regarding the participants’ ages, they range from 18 months to 23 years old. In 91.87% (n = 11) of the articles, their average age was between 18 months and 9 years, encompassing minors. Only one study included adult participants of 23 years old (Kokal et al., 2011).

#### 3.2.2. Methodological Aspects

The measurement instruments used varied depending on the studies’ objectives. In three articles, reliability information about the instruments was provided (Ilari et al., 2020; Schellenberg et al., 2015; Williams et al., 2015). Also, they explicitly indicated the details of their data availability. Cronbach’s alpha was generally acceptable (ranging from α = 0.65 to α = 0.89) for the instruments that reported it. Regarding the variables assessed, not only prosocial behavior and related factors were included. Depending on the study objectives, other variables, such as gender, vocabulary, arithmetic, participation, engagement, and attention, were examined.

The number of sessions needed was highly different among the studies. According to the length of the program, they can be classified into short (1–5 sessions, n = 6, 50%), medium (5–20 sessions, n = 3, 25%), and long (20–40 sessions, n = 2, 16.67%). Additionally, one study did not specify the number of sessions used but relied on parental information and reported musical activity criteria (Williams et al., 2015). Regarding the length of each session included in each program, they can also be classified into short (10–20 min, n = 5, 41.67%), medium (20–45 min, n = 4, 33.33%), and long (45 min to 1 h, n = 1, 8.33%). These details were not reported in two studies (Kokal et al., 2011; Williams et al., 2015). Multiple techniques and dynamics were indicated, all related to music, interpersonal synchronization, or both. These techniques included group singing, playing instruments, dancing, improvising, walking, listening to music, sound dramatization, musical montage, painting, musical notation, pentatonic scales, and performances.

Only three articles reported the effect size of their results (Buren et al., 2021; Ilari et al., 2021; Schellenberg et al., 2015). They were mainly presented using Cohen’s d or partial eta squared (*ηp*^2^). Effect size reflects the magnitude of results and provides an estimate of the scope of findings. The study by Buren et al. (2021), with values reported using Cohen’s d, presented a high-magnitude effect for general helping and spontaneous helping (general helping: d = 0.87, spontaneous helping: d = 0.94 and d = 0.96). Schellenberg et al. (2015) initially showed a medium effect size in the variable “vocabulary” (*ηp*^2^ = 0.073) and subsequently a large effect (*ηp*^2^ = 0.172). There was a large effect size in comprehension (*ηp*^2^ = 0.327 and *ηp*^2^ = 0.546). The sympathy variable “sympathy” demonstrated a medium effect size (*ηp*^2^ = 0.058, *ηp*^2^ = 0.101, and *ηp*^2^ = 0.108), and prosocial skills initially exhibited a medium effect size (*ηp*^2^ = 0.116), later increasing to a large effect (*ηp*^2^ = 0.187 and *ηp*^2^ = 0.180). Finally, Ilari et al. (2021) revealed a large effect size for helping during the pretest (*ηp*^2^ = 0.259) and a medium effect for sharing (*ηp*^2^ = 0.077).

#### 3.2.3. Observed Results

Nine articles (75%) reported positive results in terms of improving prosocial behavior among participants. For instance, Botella and Montesinos (2016) found an increase in prosocial behaviors (self-esteem, empathy, cooperation, helping, respect, and listening) in second-grade children after two weeks of music education. Similarly, Kokal et al. (2011) demonstrated that participants who played drums synchronously with a partner showed more prosocial commitment than those who played asynchronously.

#### 3.2.4. Results of CA Analysis

The AXIS questionnaire evaluation provides an overview of the bias assessment in the selected articles (Appendix C). According to this evaluation, most of the selected articles demonstrated medium quality (n = 9, 75%). Among the remaining articles, 8.33% (n = 1) showed low quality, and 16.67% (n = 2) exhibited high quality.

## 4. Discussion

A key feature of collective music creation, including music education, is participation in the synchronization of interpersonal movements (Ilari et al., 2021). The main objective of the present study was to examine whether participation in a musical interpersonal synchrony experience improves prosociality scores. Regarding this objective, a systematic review was conducted. The RQ—Which effect will the musical interpersonal synchrony have on prosociality scores among individuals?—was answered. According to these results, individuals who participated in musical interpersonal synchrony interventions improved their prosociality scores.

Prosocial behavior has also been studied through related concepts such as helping, sharing, or social skills. Naturalistic music creation, involving singing and movement between a preschool child and two unfamiliar adults, was found to create a stronger association with spontaneous helping and general sharing among participants (Buren et al., 2021). Additionally, Beck and Rieser (2022) observed that spontaneous helping was greater among 18-month-old children after participating in a musical activity. Contradicting these findings, Ilari et al. (2021) found that a short 5-week music program did not positively influence working memory control or prosocial skills such as sharing and helping.

Two of the analyzed articles reported inconsistent results. In one case, Guevara-Parra (2009) found prosociality effects only in the intervention group that received incomplete music therapy sessions. In the experimental group that received a complete music therapy intervention, no prosociality effects were observed, with only a minor improvement from low to medium scores. The author noted that although the full intervention was expected to enhance prosocial behavior, results were influenced by external factors, such as age. Additionally, the intervention showed significant effects in reducing direct aggression, including physical and verbal abuse, as well as impulsive responses to real or perceived offenses. Another study by Schellenberg et al. (2015) showed an increase in social skills, but only among children who scored poorly at the beginning of the study, not those with higher initial scores. This is a less favorable result for the review’s main objective. However, the same authors highlighted the likelihood that synchrony played an important role in prosociality.

As previously mentioned, collective music creation provides opportunities for individuals to engage in mimetic and imitative behaviors in a safe space. It fosters a sense of unity that can lead to the emergence of prosocial skills. The most frequently used techniques for creating a cooperative musical space include dancing, singing, and playing instruments (drums, ukuleles, tambourines, bells, and maracas). Music and dance are known to function as behavioral tools for mutual social bonding (Kirschner & Tomasello, 2010). Synchrony has positive effects on social bonding and cooperation in both adults (Wiltermuth & Heath, 2009) and children (Cirelli et al., 2014; Rabinowitch & Knafo-Noam, 2015; Tarr et al., 2015). While few studies have included music as a method of intervention alongside interpersonal synchronization, it is evident that interpersonal synchrony through music fosters prosociality. For instance, in the study by Buren et al. (2021), spontaneous helping increased significantly after musical activity, whereas no such effects were observed after non-musical interventions, highlighting the essential role of music in driving these improvements. In conclusion, musical activities foster the creation of an environment conducive to interpersonal synchrony activities. Music can be an effective tool for creating social spaces that encourage joint participation and prosocial development.

Regarding the second objective of the review, which seeks to identify and address the limitations causing inconsistencies in the literature, several conclusions can be drawn. First, information on effect size was evaluated. Ilari et al. (2021) presented medium and large effect sizes in the pretest for helping and sharing, indicating a significant positive effect. Buren et al. (2021) reported a large effect size for general and spontaneous helping, showing that musical intervention had a strong positive impact on this variable. They also compared active music conditions with non-musical and passive music conditions, finding a large effect size and demonstrating that children were more helpful in the active music condition. Schellenberg et al. (2015) reported medium and large effect sizes for their four variables (vocabulary, comprehension, sympathy, and prosocial skills), highlighting the positive impact of their intervention method.

Second, the number of participants was generally small. Only one article included a large sample of 3031 individuals, while the remaining studies had a lower number of participants, with more than half of the studies involving fewer than 100 participants. Such small sample sizes pose challenges in generalizing results, preventing the samples from being representative of the general population. Larger, pre-analyzed samples are needed to ensure representativeness and avoid study limitations.

Third, potential links between music education and prosocial behavior have generally been studied in children (Ilari et al., 2020). The predominance of studies on children suggests that favorable results in prosociality should be interpreted for this population. Fourth, all studies with a single intervention session found improvements in prosociality scores. Less favorable results were observed in studies with a larger number of sessions. One hypothesis for the inverse relationship between the number of sessions and poorer results is that more frequent sessions may lead to participant fatigue, reducing engagement and motivation. Additionally, repeated exposure to the same intervention could cause habituation, making participants less responsive over time. Other variables, such as age, intervention type, and sample size, may influence these outcomes and should be studied further. Thus, even a single musical interpersonal synchrony session during childhood has been shown to improve prosociality.

Fifth, gender differences in prosociality were noted, with girls generally scoring higher in tasks related to helping others. These gender implications are discussed from social role, cultural context, and developmental trajectory perspectives (Wan & Zhu, 2021). Kirschner and Tomasello (2010) found that girls help and cooperate more than boys. This aligns with Wan and Zhu’s (2021) findings, who observed that girls were more likely to help their partner in block assembly tasks. However, boys acted more generously than girls in a task that involved sharing. This contradiction aligns with Wan and Fu’s (2019) research, which showed that boys displayed greater cooperation in a cooperative resolution task after synchronous instrument playing. The plurality of results suggests no clear gender differences in prosocial behavior.

### Limitations and Future Research

This study is not absent of limitations. On the one hand, it has excluded the articles that assessed interpersonal synchrony and prosociality without musical interventions. Literature shows favorable outcomes in interpersonal synchrony and prosociality without music. Investigating and comparing these effects in future reviews and creating new studies would be valuable.

On the other hand, the scope included the general population. While the present study aimed to maximize article inclusion by not setting age limits, studies on children dominate this field and show predominantly positive effects. Narrowing the focus to children could offer more specific insights into the effects of interventions on this population, making it a promising area for further research into prosociality development.

Furthermore, the methods that were used throughout the studies varied widely. There was insufficient data to corroborate the reliability and validity of all the measurement tools used. Numerous different instruments assessed variables such as demographics, skills, prosocial behavior, sympathy, cooperation, helping, attention, sharing, and vocabulary, as detailed in the results table. A significant portion of the instruments relied on tasks like block stacking, bead sorting, envelope handling, or clothespin tasks. These tasks were specifically created by researchers to measure variables and were used in previous studies, implemented by professionals. However, the diversity of instruments and variables assessed represents a limitation. Ensuring the reliability of tools is crucial, enabling researchers to use the most effective tools in future studies. Establishing consensus on the use of instruments and selecting optimal variables is essential to ensure result quality. In the same line, the number of intervention sessions varied widely, ranging from single sessions to 40 session. That prevented the researchers from ensuring a generalization of the results derived from the studies included.

Additionally, limiting the inclusion criteria to articles in English or Spanish within selected databases may have excluded other relevant studies. Similarly, using only seven databases (WoS, Scopus, PubMed, Dialnet, Elsevier, and Eric) could mean relevant articles from other sources were not considered. Also, the publication bias is always a limitation that must be considered regarding a systematic review.

Future research must address the limitations identified in this review, including sample size and age, measurement instruments, sessions, and intervention techniques. For example, the fact that only one article focused on adults. Also, given the greater number of studies and their predominantly positive effects, childhood appears to be a more accessible area for future research. It is assumed to be a promising field for teaching prosocial behavior. Another potential line of study could explore including participants with psychological disorders, as this may provide valuable insights into how interventions can be tailored for individuals with specific psychological needs. These aspects should be refined for future studies examining the relationship between musical and interpersonal synchrony interventions and prosociality improvement. Recognizing study limitations will help guide future research.

The findings of this study have valuable implications not only for researchers but also for professionals working in educational settings. To optimize interventions aimed at promoting prosocial behavior, it will be important to examine how different factors, such as session frequency, duration, and structure, influence participant engagement and outcomes. Additionally, the role of the teacher, particularly in fostering an environment of autonomy and motivation, could be critical for the success of such interventions. Future research should also explore how various social contexts affect the effectiveness of these interventions and identify specific behaviors that may facilitate or hinder prosocial development.

## 5. Conclusions

This review aimed to confirm music’s role as a facilitator of interpersonal synchrony activities, promoting group interaction. The results suggest the practical benefits of using music, reinforcing its potential as a universal tool for social connection. The findings highlight the promising field of childhood education in prosociality, emphasizing music and interpersonal synchrony as key components. From a broader perspective, ethnomusicology underscores music’s universal nature, transcending cultural and social boundaries to foster interaction, empathy, and shared experiences (Lin et al., 2023). This study aligns with this view, demonstrating how musical participation and synchrony contribute to prosocial behaviors by creating inclusive spaces where individuals engage through mimicry and collective methodologies (Thaut et al., 2021; Tramontano et al., 2021). However, given the scarcity of scientific literature on the review’s main objective and the favorable outcomes in this research area, further comparative studies are necessary to address the limitations identified in this work. Nevertheless, the results highlight promising effects in the areas of music, interpersonal synchrony, and prosociality, reinforcing the idea that music is not only an artistic expression but also a powerful medium for social cohesion and cultural exchange (Campbell, 2020; Crawford, 2020).

## Figures and Tables

**Table 1:** Characteristics of the included studies.

Authorship, Publication Year and Country	Measures	Variables	Sample N Age (Min.–Max. Years Old) Brief Description	Effect Size	Session’s Description	Techniques and Dynamics	Results
Guevara-Parra (2009) Colombia	COPRAG Standardized Emotional Disorders Battery	Antisocial behavior PB Emotional disorders	18 (5–9) Preschool children at risk of aggression	NR	Frequency: 30 sessions. Duration: 20 min Phases: Initial evaluation, phase 1, and phase 2 interventions	Synchronization in group singing, musical montage, and improvisation. Techniques: Music therapy, instrumental improvisation, group singing, sound dramatization, painting to music, body as an instrument, singing dances, movement improvisation, role-playing, music interaction games	Music therapy had a significant effect only on the PB of the group receiving incomplete intervention, improving from low to medium
Kokal et al. (2011) The Netherlands	Social Commitment Test Experimental Questionnaire	Commitment Helping Satisfaction	18 (19–30) Healthy female volunteers without musical training	NR	4 sessions. Phases: Training session of familiarization with synchronized rhythm, fMRI scanning session, fMRI testing session	Playing drums. Synchronization and non-synchronization while drumming	Participants who played drums synchronously with a partner showed higher prosocial commitment than those playing asynchronously
Kirschner and Tomasello (2010) Germany	Spontaneous Helping Task Spontaneous Cooperative Problem-Solving Task	Spontaneous Cooperative Problem-Solving Voluntary Helping	96 (4–5)	NR	Frequency: One session. Duration: 20 min. Phases: two experimental manipulation phase	Musical condition: Walking in synchronization, singing a song, playing instruments in synchronization with lyrics. Non-musical condition: Walking and crawling, objects moving at synchronized and non-synchronized intervals	Joint music-making in sync improved PB in 4-year-old children, demonstrating that music and dance serve as behavioral tools for mutual social bonding
Schellenberg et al. (2015) Canada	Peabody Picture Vocabulary Test (4th Ed.) Emotion Comprehension Test Subscales Child-Report Sympathy Scale Social Behavior Questionnaire	Vocabular Comprehension Sympathy Social Skills	84 (8–10) Public school students	Vocabulary: *ηp*^2^ = 0.073 *ηp*^2^ = 0.172. Comprehension: *ηp*^2^ = 0.327 *ηp*^2^ = 0.546. Sympathy: *ηp*^2^ = 0.058 *ηp*^2^ = 0.101 *ηp*^2^ = 0.108. Prosocial Skills: *ηp*^2^ = 0.116 *ηp*^2^ = 0.187 *ηp*^2^ = 0.180	Weekly sessions over 10 months (school year). Duration: 40 min	Synchrony during singing exercises and playing the ukulele. Techniques: Hand signals, auditory training, musical notation, pentatonic and diatonic scales, solo and group performances, improvisation	Social skill improvement was observed only in children who initially scored poorly. Synchrony likely played a key role in prosocial development
Williams et al. (2015) Australia	Home Music Activities Scale Home Reading Scale Peabody Picture Vocabulary Test (3rd Ed.) Numeracy Scale Self-Concept Questionnaire Short Temperament Scale for Children Subscale of Strengths and Difficulties Questionnaire	Prosocial Skills Self-Regulation. Emotional Regulation Arithmetic Vocabulary School Readiness Behavioral Problems	3031 (4–5)	NR	No intervention sessions. Data derived from mothers’ reports	Music activities: Dance participation, action songs, playing instruments, activities involving close proximity, and shared actions	Shared musical activities were strongly associated with children’s subsequent prosocial skills
Botella and Montesinos (2016) Spain	Selective Attention Test Divided Attention Test Sustained Attention Test Sociometric Questionnaire for Students Sociometric Questionnaire for Educators Direct Observation PB Questionnaire Teacher and student responses	Attention: divided, sustained, selective PB: parameters of self-esteem, empathy, cooperation, helping, respect and listening	24 (7–8)	NR	Frequency: 7 sessions over two weeks. Duration: 45 min. Phases: Pre-test phase: 1 session; intervention phase: 5 sessions; post-test phase: 1 session	Cooperative, helping, empathetic, and self-esteem-building musical activities	Improvements in respect, listening, helping, and cooperation were noted, along with slight gains in self-esteem and empathy. Music education contributed to better attention and prosocial behavior in education
Wan and Fu (2019) China	Cooperative Problem-Solving Helping Task	Cooperative Problem-Solving Spontaneous Helping Rhythm Predictability Rhythmic Consistency	124 (5–6)	NR	Frequency: One session. Duration: 20 min. Phases: Procedure demonstration for children: 30 s; semi-guided game: 1 min; guided game: 1 min; percussion game	Music creation using instruments. Synchronized music-making following a marked pattern	Percussion with uniform rhythms increased PB in 5-year-olds. Boys were more cooperative in the apron task, while girls scored higher in helping tasks. Results suggest prosocial effects of interpersonal coordination are influenced by rhythmic consistency
Ilari et al. (2020) The USA	Block Game: Helping and Sharing Participation Index Prosociality Index Demographic and Musical Interest Questionnaire	Prosociality Active Participation Instrumental Helping Sharing	36 (37–56 months) Children enrolled in early childhood programs for 1–54 months	NR	Frequency: 10-week modules, weekly music classes for ages 0–8 years accompanied by an adult. Duration: 1 h. Group size: up to 12 children	Singing, movement and dancing. Music listening. Music creation. Improvisation. Playing instruments. Activities indicated coordination and group collaboration	Children’s responses to prosocial tasks varied qualitatively. Children who spent more time in the music program performed better in instrumental helping tasks. Parental ratings of higher prosociality correlated with children’s active musical engagement
Wan and Zhu (2021) China	Likert Mood Scale Helping Task Sharing Task	Mood Sharing	108 (5–6) Children from a rural town in China	NR	Frequency: One session. Duration: 20 min. Phases: Manipulation phase; music play: Playing “Jingle Bells” with an instrument (drum or bell)	Playing instruments. Coordination in joint music-making	Joint music-making coordination influenced prosocial behaviors in 5-year-olds. Fine-grained coordination facilitated early collaboration and socialization. The effects generalized to unfamiliar individuals. Girls were more likely to perform helping tasks, while boys were more generous in sharing tasks
Ilari et al. (2021) The USA	Helping Game Dimensional Change Card Sort Spin Pots Task Peabody Picture Vocabulary Test (PPVT-4)	Instrumental Helping Sharing Cognitive Flexibility Working Memory Inhibitory Control Vocabulary	103 (4–6)	Pretest Helping: *ηp*^2^ = 0.259. Sharing: *ηp*^2^ = 0.077	Frequency: 5 weeks, twice per week. Duration: 40 min. Phases: the experimental group received music classes while the control group practiced collective activities	Singing Action songs Playing instruments Movement activities Songs and instrumental music with props Relaxation activities Coloring Crafts Small group projects	No significant differences in instrumental helping scores pre- and post-test. Children with lower initial prosocial scores for helping and sharing showed improvements post-test. A 5-week music program positively influenced cognitive flexibility but not working memory control or prosocial skills
Buren et al. (2021) Germany	Clothespin Task Marker Task Paper Ball Task Social, Economic and Temperament Questionnaire	Helping: General, Spontaneous and Delayed	50 (18 months)	General Helping: d = 0.87 d = 0.93 d = 0.86. Spontaneous Helping: d = 0.96 d = 0.94.	Frequency: One session. Duration: 45 min. Phases: warm-up phase: 2 min playing ball; intervention phase: 4 min; test phase: evaluation	Music creation Instrument playing Singing Conditions: Active Music: Musical representation of a song with a shaker; Passive Music: Listening to a song with a manipulated shaker. Non-Musical Activity: Looking at a booklet and reciting parts of the song	Active music-making significantly increased spontaneous helping behavior compared to non-musical activities. Joint music creation facilitated PB
Beck and Rieser (2022) The USA	Sharing Task Helping Task Understanding Verification	Helping Sharing Content Understanding	62 (47–75 months)	NR	Frequency: One session. Duration: 10–13 min. Phases: Consent and warm-up period; introduction by Research Assistant and puppets; experimental interaction	Musical Condition: Singing, swaying, playing maracas while singing. Non-Musical Condition: Reciting the song, laying maracas	Naturalistic music-making, including singing and movement with preschoolers and two unfamiliar adults, was associated with increased spontaneous helping and general sharing compared to non-musical play. Joint music-making influenced subsequent PB and interaction

Note. PB: Prosocial behavior; NR: Not reported.

## Data Availability

Data availability is not applicable to this article as no datasets were generated or analyzed during this study.

## Abbreviations

The following abbreviations are used in this manuscript:

PB	Prosocial behavior
RQ	Research question
PRISMA	Preferred Reporting Items for Systematic reviews and Meta-Analyses
PROSPERO	International prospective registers of systematic reviews
WoS	Web of Science
AXIS	Appraisal tool for Cross-Sectional Studies
CA	Critical appraisal

## Appendix A

PRISMA checklist and the page in which each item appears in the text.

**Items**	**No**	**Yes**	**Page**	**NA**
TITLE				
1. Title		X	1	
ABSTRACT				
2. Structured abstract		X	1	
INTRODUCTION				
3. Rationale		X	1	
4. Objectives		X	4	
METHODS				
5. Eligibility criteria		X	5	
6. Information sources		X	4	
7. Search strategy		X	4	
8. Study selection process		X	5	
9. Data extraction process		X	5	
10. List of data		X	5	
11. Risk of bias		X	5	
12. Effect measures				X
13. Synthesis methods				X
14. Assessment of publication bias				X
15. Certainty assessment				X
RESULTS				
16. Study selection		X	6	
17. Study characteristics		X	6	
18. Risk of bias		X	14	
19. Results of individual studies		X	7	
20. Results of synthesis		X	10	
21. Publication bias				X
22. Certainty of evidence		X		
DISCUSSION				
23. Discussion		X	17	
OTHER INFORMATION				
24. Registration and protocol		X	4	
25. Funding		X	21	
26. Conflict of interest		X	21	
27. Availability of data				X
Note. NA: Not applicable.				

## Appendix B

AXIS tool items

**Introduction**
1. Were the purposes/objectives of the study clear? Methods 2. Was the study design appropriate for the stated objectives? 3. Was the sample size justified? 4. Was the target/reference population clearly defined? (Is it clear who the research was about?) 5. Was the sampling frame taken from a population base appropriate to faithfully represent the target/reference population under investigation? 6. Was the selection process likely to recruit subjects/participants representative of the target/reference population under investigation? 7. Were measures taken to address and categorize non-responders? 8. Were the risk factors and outcome variables measured appropriately for the objectives of the study? 9. Were the risk factors and outcome variables properly measured using instruments/measures that had been previously tested, validated, or published? 10. Is it clear what was used to determine statistical significance and/or precision estimates? (e.g., p-values, CIs) 11. Were the methods (including statistical methods) sufficiently described to allow replication? Results 12. Were the baseline data adequately described? 13. Does the response rate raise concerns about non-response bias? 14. If applicable, was information about non-responders described? 15. Were the results internally consistent? 16. Were the results of the analyses described in the methods presented? Discussion 17. Were the authors’ discussions and conclusions justified by the results? 18. Were the study’s limitations discussed? Other 19. Were there any sources of funding or conflicts of interest that could affect the authors’ interpretation of the results? 20. Was ethical approval or participant consent obtained?
Note: Quality Scoring: Low = < 12.5 points, Medium = 12.5–15, High > 15 points.

## Appendix C

Results for the AXIS tool questionnaire for the studies included in the review.

**Studies**	**AXIS Tool Questions**																				**Total**	**Quality**
	**1**	**2**	**3**	**4**	**5**	**6**	**7**	**8**	**9**	**10**	**11**	**12**	**13**	**14**	**15**	**16**	**17**	**18**	**19**	**20**		
Guevara-Parra (2009)	Yes	Yes	Yes	Yes	Yes	Yes	No	Yes	Yes	No	Yes	Yes	No	No	Yes	Yes	Yes	Yes	No	NM	14	Medium
Kokal et al. (2011)	Yes	Yes	No	Yes	Yes	Yes	No	Yes	Yes	No	Yes	Yes	No	No	Yes	Yes	Yes	Yes	No	Yes	15	Medium
Kirschner and Tomasello (2010)	Yes	Yes	Yes	Yes	Yes	Yes	Yes	Yes	Yes	Yes	Yes	Yes	No	No	Yes	Yes	Yes	No	No	No	16	High
Schellenberg et al. (2015)	Yes	Yes	No	Yes	Yes	Yes	No	Yes	Yes	No	Yes	Yes	No	No	Yes	Yes	Yes	No	No	Yes	14	Medium
Williams et al. (2015)	Yes	Yes	No	Yes	Yes	Yes	Yes	Yes	Yes	Yes	Yes	Yes	No	No	Yes	Yes	Yes	No	No	No	15	Medium
Botella and Montesinos (2016)	Yes	Yes	No	Yes	Yes	Yes	No	Yes	Yes	No	Yes	Yes	No	No	Yes	Yes	Yes	No	No	No	13	Medium
Wan and Fu (2019)	Yes	Yes	Yes	Yes	Yes	No	No	Yes	Yes	No	Yes	Yes	No	No	Yes	Yes	Yes	No	No	Yes	14	Medium
Ilari et al. (2020)	Yes	Yes	No	Yes	Yes	No	No	Yes	Yes	No	Yes	Yes	No	No	Yes	Yes	Yes	Yes	No	No	13	Medium
Wan and Zhu (2021)	Yes	Yes	No	Yes	Yes	No	No	Yes	Yes	No	Yes	Yes	No	No	Yes	Yes	Yes	No	No	No	12	Low
Ilari et al. (2021)	Yes	Yes	No	Yes	Yes	No	Yes	Yes	Yes	No	Yes	Yes	No	No	Yes	Yes	Yes	Yes	No	No	14	Medium
Buren et al. (2021)	Yes	Yes	Yes	Yes	Yes	Yes	No	Yes	Yes	Yes	Yes	Yes	No	No	Yes	Yes	Yes	Yes	No	Yes	17	High
Beck and Rieser (2022)	Yes	Yes	Yes	Yes	Yes	Yes	Yes	Yes	Yes	Yes	Yes	Yes	No	No	Yes	Yes	Yes	Yes	No	No	17	High
Note. NM: Not measured.																						
